# Development of a Shock and Detonation Velocity Measurement System Using Chirped Fiber Bragg Gratings

**DOI:** 10.3390/s20041026

**Published:** 2020-02-14

**Authors:** Yohan Barbarin, Alexandre Lefrançois, Vincent Chuzeville, Sylvain Magne, Laurent Jacquet, Thomas Elia, Karol Woirin, Christelle Collet, Antoine Osmont, Jérôme Luc

**Affiliations:** 1 CEA, DAM, GRAMAT, BP 80200, F-46500 Gramat, France; Alexandre.LEFRANCOIS@CEA.FR (A.L.); Vincent.CHUZEVILLE@CEA.FR (V.C.); Laurent-N.JACQUET@cea.fr (L.J.); thomas.elia@gadz.org (T.E.); Antoine.OSMONT@CEA.FR (A.O.); Jerome.LUC@CEA.FR (J.L.); 2 CEA, LIST, Laboratoire Capteurs Fibres Optiques, F-91191 Gif-sur-Yvette, France; Sylvain.MAGNE@cea.fr; 3 Formerly at ArianeGroup\CRB, F-91710 Vert-le-Petit, France; karol.woirin@mbda-systems.com (K.W.); c.collet@msiac.nato.int (C.C.)

**Keywords:** fiber Bragg grating (FBG), chirped FBG, high explosive, shock, detonation, velocity measurement

## Abstract

Dynamic measurements of shock and detonation velocities are performed using long chirped fiber Bragg gratings (CFBGs). Such thin probes, with a diameter of typically 125 µm or even 80 µm can be directly inserted into high-explosive (HE) samples or simply glued laterally. During the detonation, the width of the optical spectrum is continuously reduced by the propagation of the wave-front, which physically shortens the CFBG. The light power reflected back shows a ramp-down type signal, from which the wave-front position is obtained as a function of time, thus yielding a detonation velocity profile. A calibration procedure was developed, with the support of optical simulations, to cancel out the optical spectrum distortions from the different optical components and to determine the wavelength-position transfer function of the CFBG. The fitted slopes of the X–T diagram give steady detonation velocity values which are in very good agreement with the classical measurements obtained from discrete electrical shorting pins (ESP). The main parameters influencing the uncertainties on the steady detonation velocity value measured by CFBG are discussed. To conclude, different HE experimental configurations tested at CEA (*Commissariat à l’Energie Atomique et aux Energies Alternatives*) are presented: bare cylindrical sticks, wedges for shock-to-detonation transitions (SDT), spheres, a cast-cured stick around a CFBG, and a detonation wave-front profile configuration.

## 1. Introduction

**Figure 1:**
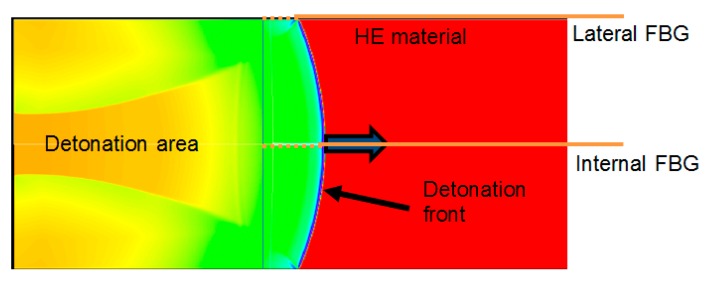
Possible fiber Bragg grating (FBG) positioning in a high-explosive (HE) material cylinder for detonation velocity measurement. The colored background is the result of a hydrodynamic simulation.

**Figure 2:**
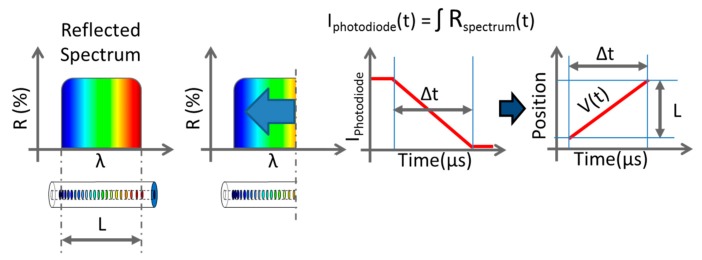
Detonation velocity measurement using a chirped FBG (CFBG). In the reflected spectrum of the CFBG, a wavelength corresponds to a position along the fiber. When the fiber is shortened by the detonation, the intensity of the reflected light decreases linearly with time. Thanks to a proper calibration, this intensity corresponds to a position and then a velocity can be deduced.

Chirped fiber Bragg gratings (CFBGs) are now commonly used for continuous and dynamic detonation wave-front location measurements in high-explosive (HE) materials with recording rates higher than 10 MHz. Initially, a few detonation velocity measurements were reported using bare optical fibers [1,2] by the Lawrence Livermore National Laboratory (LLNL) and the Los Alamos National Laboratory (LANL). The system was based on the Doppler effect of the reflected optical signal at the bow shock inside the fiber attached to the detonation wave-front. However, this technique requires a high-power laser and is very sensitive to the wave-front shape, which is the case when the wave-front velocity is higher than in silica. On the other hand, CFBGs offer very good signal-to-noise ratio (SNR); moreover, they allow versatile configurations, and the optical power needed can be limited to a Class 1 optical source near 1550 nm for both pyrotechnic and laser safety considerations. Many groups in the United States (LLNL, Columbia Gorge Research, and LANL), France (CEA), Israel (NRC Soreq), China (BUAA), and England (AWE + ORC Southampton) reported results [3,4,5,6,7,8,9,10,11,12,13,14,15,16,17,18,19,20] over the last two decades. The measurement principle remains the following: a CFBG is either placed along or even inside an HE material as shown in Figure 1. Along or inside, the detonation velocities would be the same if steady detonation is reached, which is the case at the end of the stick. As the detonation wave-front propagates along the HE material, the CFBG is highly shocked or even destroyed, depending on the detonation pressure. The system is suitable for high shock and detonation wave-fronts. As a result, the power reflected back by the CFBG gradually drops from a level “1” to a level “0”, as illustrated in Figure 2. The position of the detonation or shock wave-front along the fiber corresponds to a wavelength on the CFBG reflected spectrum, and it needs to be carefully calibrated beforehand.

Early on, the calibration, i.e., the amplitude of the CFBG reflecting signal through the complete measurement system as a function of its length, was carried out in numerous steps by cutting the CFBG with a laser [3,7] or by polishing it [9,12]. Another method, non-destructive, was to characterize the linearity of the chirp as a function of the position using a local heating technique thanks to a small tip touching the CFBG [4,7,9]. This tip is shifted step by step along the grating, and its location is visible as a dip in the reflected optical power. From this second method, the chirp rate (CR) of the CFBG is extracted and the fiber response calculated from the shape of the optical source spectrum [4,7,12].

We propose a novel calibration method based on OFDR (optical frequency-domain reflectometry). It was very briefly introduced in Reference [14], and it is extensively presented in Section 4. It makes use of a Luna OBR 4600 equipment and has the advantages of being non-destructive, fast, and accurate in the space domain (0.01 mm). In return, the spatial uncertainty is conditioned by the uncertainty concerning the value of the effective index *n_eff_* of the guided mode, as the OBR 4600 measured the amplitudes of the reflected wavelength with respect to optical distance [21].

Knowing the CFBG’s CR is not entirely sufficient to obtain the complete CFBG response as a function of the position. To be more accurate, one needs to carefully analyze the CFBG response at the beginning and at the end of the grating. We mentioned in Reference [12] that the response of the CFBG is typically usable with a seamless linear response in the range of 5% up to 95%. Very recently, Pooley et al. provided recommendations in Reference [20] on the CFBG design to maximize this CFBG response range. A parallel approach is presented in Section 3 with optical simulations and in Section 4 with experimental tests.

Any given measurement value should come with an uncertainty estimation. In our particular topic of detonation velocity measurements using CFBG, Wei et al. initiated a study in Reference [18] and announced an uncertainty on the detonation velocity values below 1%. We propose in Section 5 of this paper a novel theoretical approach taking into account the signal processing.

To complete this short overview on the use of CFBG in detonation velocity measurements, we can also mention that uniform fiber Bragg gratings (FBGs) can also be used in other ways to measure detonation velocities. CEA reported in Reference [22] the use of a series of short uniform Bragg gratings along a single-mode fiber. The temporal response obtained has a staircase shape. The positions of the individual FBGs are also measured with an OFDR. The signal processing consists of detecting the jumps between each plateau in the signal. This approach requires a simpler signal processing; however, the measurement is not continuous. Very recently, the use of a single uniform FBG was demonstrated by Pooley et al. [23]. The grating is 10 mm long thanks to a limited refractive index modulation. The grating peak reflectivity decreases as a function of the wave-front position. The grating response is not perfectly linear and needs to be carefully calibrated. The grating response is better if the maximum reflectivity is below 50%. One limitation might be that, to make longer sensors (>50 mm), the refractive index modulation reduction might reach a limit. The same year, the same authors reported in Reference [24] the use of rare-earth doped fibers. The sensor section of the fiber is doped with erbium and ytterbium ions and is pumped like an erbium-doped fiber amplifier (EDFA). The detonation wave-front destroys the active fiber and the amplified spontaneous emission (ASE) signal decreases in a proportional amount. One advantage is that very long sensors could be created; however, the calibration is not easily reproducible from one fiber to another because it is very dependent on the low pump power to get a response as linear as possible.

Different HE material experimental configurations were tested at CEA Gramat and at CRB (*Centre de Recherche du Bouchet*, ArianeGoup) over the last decade [9,12,14,15,16,17,25]. New experimental results, with uncertainty calculations, are presented in Section 6 of this paper: bare cylinder, wedges for shock-to-detonation transitions, and sphere configurations. An experiment of a cast-cured stick around a CFBG [15] is also described with an updated signal processing to provide uncertainty boundaries on the steady detonation value. Finally, a detonation profile configuration is shown with further information on the integration of the fiber than provided in Reference [17].

## 2. BraggFast System

**Figure 3:**
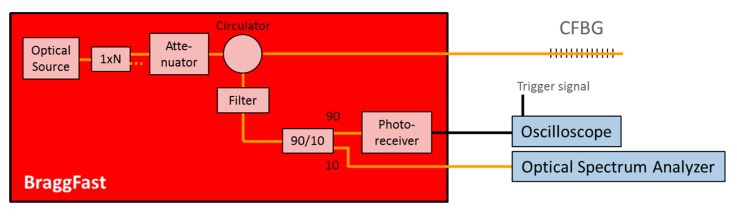
BraggFast system for detonation velocity measurements using a CFBG.

The BraggFast is the CFBG interrogation system for detonation velocity measurements. The block diagram of the system is depicted in Figure 3. The common optical source is an ASE or a super-luminescent diode (SLED). Depending on the number of measurement channels, the source is split and the following list of optoelectronic parts is duplicated. The maximum optical power sent to a CFBG is limited to 10 mW; thus, the laser system remains in Class 1. A circulator routes the reflected signal from the CFBG to a coupler with a 90%–10% ratio. Most of the power (90%) is collected by the fast photoreceiver with a bandwidth higher than 250 MHz, while 10% is routed to a standard optical spectrum analyzer (OSA) to measure the initial optical spectrum. An attenuator is used to adjust, if needed, the photoreceiver signal levels in the same range just below saturation when different types of CFBG are used. The photoreceiver is connected to a real-time oscilloscope, which is triggered by the igniter of the detonator. The OSA is used for only a few minutes before the experiments; the calibration procedure is presented in Section 4.

## 3. Optical Simulation of the CBFG during Detonation

In order to better understand the limits in the CFBG response, a series of optical simulations were carried out. The simulations were performed using the field-transfer matrix theory [26]. Since all Bragg pairs were simulated, it requires high computer memory but it has the great advantage of properly simulating any asymmetric design and any abrupt change in refractive index compared to analytical equations [27]. A beam propagation method (BPM) [28] could have been used as well. The following input parameters used in the simulations are very close to those used for the longest CFBG design used in our experiments:

- Central wavelength: 1545 nm;
- Length: 100 mm;
- Chirp rate: 0.16 nm/mm;
- Averaged refractive index: 1.47;
- Refractive index modulation: 2.5 × 10^−4^;
- Apodization: with and without.

**Figure 4:**
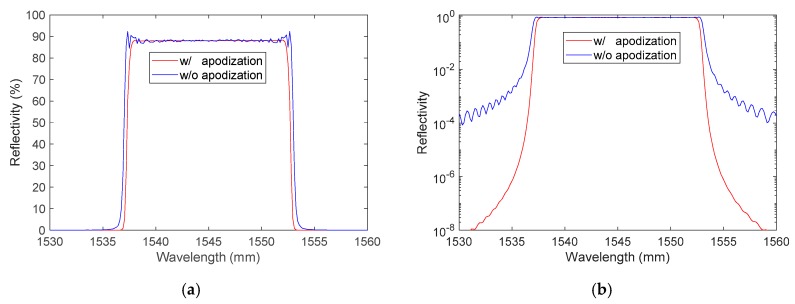
Simulated reflected optical spectrum of a 100-mm-long CFBG with and without apodization in a linear scale (**a**) and a logarithmic scale (**b**).

The simulated optical spectra with and without apodization (sinusoidal shape), in linear and logarithmic scale, are plotted in Figure 4a,b. The following characteristics were obtained without apodization:

- Center maximum reflectivity: ~88%;
- Spectrum full width at half maximum: 16.2 nm.

The following characteristics were obtained with apodization:

- Center maximum reflectivity: ~88%;
- Spectrum full width at half maximum: 15.6 nm.

As expected, the reflected optical spectrum with apodization had no oscillation on the top of the spectrum and had very limited side lobes visible in the logarithmic scale plot. The spectral bandwidth was slightly smaller. However, this could have an effect on the fiber response. Furthermore, as soon as the CFBG was shortened, the design remained apodized only on one side. The usefulness of apodization was then questionable.

**Figure 5:**
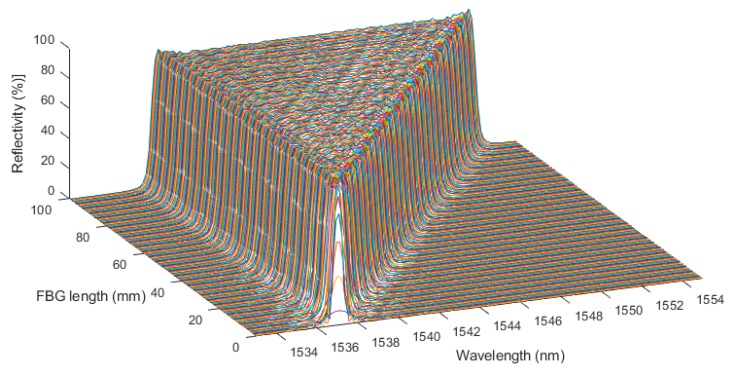
Simulation of the shortening of the 100-mm-long CFBG without apodization.

**Figure 6:**
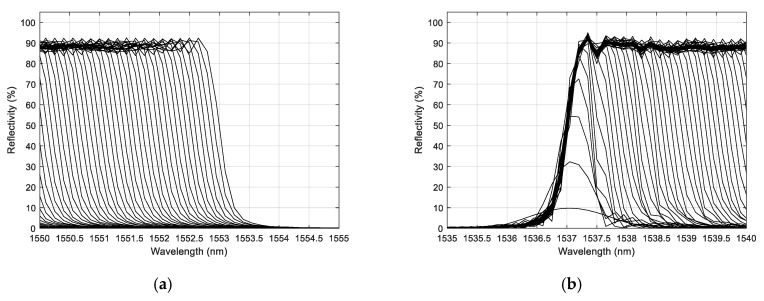
A closer look at the beginning (**a**) and the end (**b**) of the shortening process of the 100-mm-long CFBG without apodization.

The shortening of the CFBG, as happens during detonation, was then simulated in 200 steps. All the simulated spectra for a 100-mm-long CFBG without apodization are plotted in Figure 5. The spectral width seemed very linear with the length of the CFBG. It is interesting to look at the beginning and the end of this process. The spectra are replotted with a 5-nm span in Figure 6a,b respectively centered on 1552.5 nm and 1537.5 nm. When the shortening process started, since the CFBG was not apodized, the reduction of the optical bandwidth was immediate, as if the edge was just shifted. The phenomenon was different at the end of the shortening process. When the CFBG started to be less than 1.5 mm, the maximum reflectivity was not reached, and the optical spectrum was even broadened. The decrease in the reflected amplitude was less likely to be linear.

**Figure 7:**
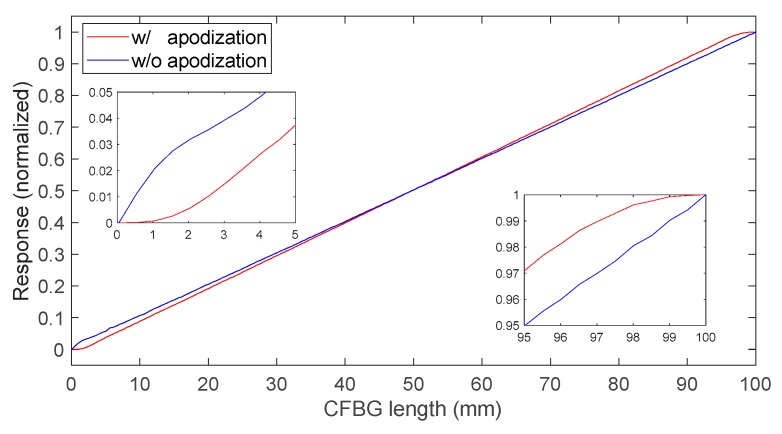
Comparison of CFBG intensity response, calculated by integrating the spectra one by one, as a function of the CFBG length, with and without apodization.

The CFBG without apodization response was calculated by integrating the previously calculated spectra one by one. The same calculations were performed for a series of spectra in the case of an apodized design. The first spectrum amplitude value of each series was used to normalize the following ones. The normalized CFBG responses as a function of the CFBG length were then obtained; the results are plotted in Figure 7 with and without apodization. As discussed in Reference [12] and more recently in Reference [20], apodization is pointless for this application. It mostly reduces the sensitivity of the system over the first few millimeters of the CFBG facing the detonation wave-front. When the fiber was very short, with or without apodization, the sensitivity was reduced. In the case of the non-apodized design, as shown in Figure 6b, the CFBG spectrum changed in amplitude and shape. In the case of the apodized design, the same phenomenon occurred and, in addition, the refractive index change was gradually reduced. Over 90% to 95% of the CFBG length, the CFBG response was perfectly linear as expected.

Such a response is needed to process an experimental BraggFast signal. However, since the ASE source, the optoelectronic components of the BraggFast system, and even the CFBG samples are not ideal, this response has to take into account all spectral distortions. A direct solution to obtain this response is to assume that all CFBGs from a same batch are identical and to sacrifice a CFBG by cutting it step by step with a laser [3,7] or by polishing it [9,12], while recording the reflected amplitude directly on the BraggFast system. A better way, which is used at CEA, is to process the initial reflected spectrum of the CFBG. Indeed, knowing the CR, the CFBG response can be calibrated even with the sensor already in place in an experimental configuration. This is a great advantage as detonic experiments are performed in environments very different from an air-conditioned laboratory. Details on this calibration are provided in the next section.

## 4. Calibration Procedure Using the Initial Reflected Spectrum

### 4.1. Theoretical CFBG Response

The CFBG response can be obtained from the reflected spectrum in the BraggFast system. Indeed, one wavelength corresponds to one position along the CFBG, and, since the CR is constant, the relation is linear by design. When a CFBG is shortened, the reflected spectrum of the remaining part of the CFBG does not change significantly, as shown in Figure 6a. Then, all spectral distortions are multiplied in the wavelength domain and can be measured from the CFBG reflected spectrum with a calibrated OSA. In our system, 10% of the reflected spectrum was sampled (see Figure 3). The spectral distortions of the 90:10 coupler were measured individually and taken into account in our signal processing tool. The BraggFast system measured the amplitude of the reflected spectrum, and this was also the integral of the spectral density as a function of the wavelength. If the CFBG is shortened, the end of the CFBG corresponds to a wavelength. The normalized CFBG response in the wavelength domain can be written as in Equation (1).

(1) $$\mathrm{RESP}\left(\lambda \right)=\frac{\int_{\lambda_{0}}^{\lambda }Initial\_Spectrum\left(\lambda \right)\cdot d\lambda }{\int_{\lambda_{0}}^{\lambda_{end}}Initial\_Spectrum\left(\lambda \right)\cdot d\lambda }.$$

Then, knowing the CR of the CFBG, this response could be calculated as a function of the CFBG length by a linear change of axis.

### 4.2. Simulated CFBG Response from the Initial Spectrum

**Figure 8:**
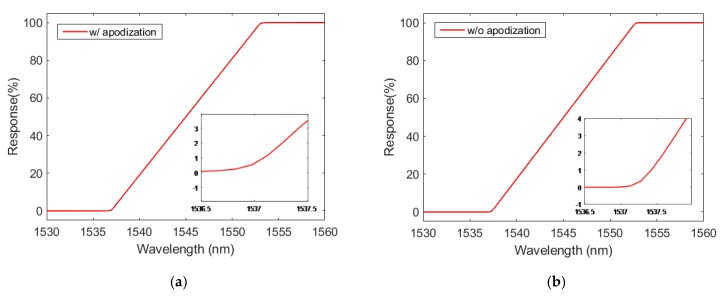
CFBG response (in %) as a function of the wavelength by integrating the initial spectrum: (**a**) design with apodization; (**b**) design without apodization.

The objective of this section is to numerically confirm that the CFBG response could be calibrated from the initial CFBG spectrum. The spectra simulated in Section 3 were reused. The first step consisted of integrating the initial spectrum as a function of the wavelength using Equation (1). Figure 8 presents CFBG responses as a function of wavelength. The responses increased with the wavelength as in Section 3, where the longer wavelengths faced the shock or the detonation wave at the end of the fiber. The design without apodization showed sharper corners near the levels “1” and “0”.

**Figure 9:**
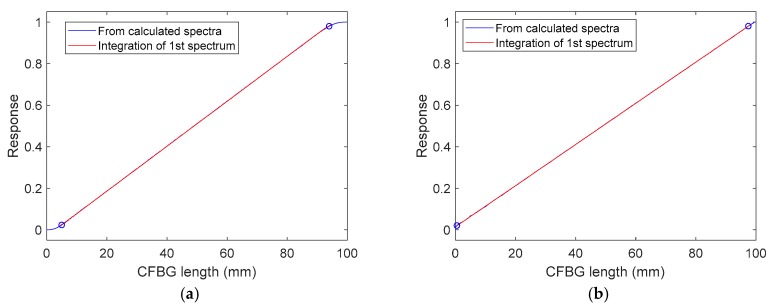
CFBG response as a function of the CFBG length by integrating the initial spectrum fitted on the CFBG response calculated with spectra as a function of the CFBG length: (**a**) design with apodization; (**b**) design without apodization.

To assess the linearity of these slopes between the 2% and 98% response in amplitude, they were both fitted in order to obtain the same CFBG responses as a function of its length as calculated in Figure 7 with all the spectra. The linear coefficient obtained was inversely proportional to the CR. The results are plotted in Figure 9: the correlation coefficients were better than 0.9999 for both designs. For the non-apodized and apodized designs, the fitted CRs were 0.1599 and 0.1600 nm/mm, respectively. This proves that, theoretically, we could obtain the CFBG response from the initial optical spectrum.

**Figure 10:**
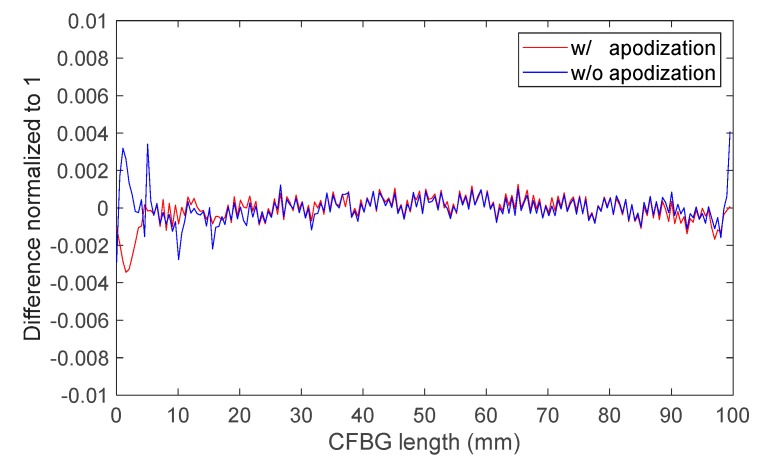
Difference between the CFBG response calculated from the initial optical spectrum and that calculated with spectra as a function of CFBG length.

If we plot the complete response (even near “1” and “0”) as a function of the wavelength by integrating the initial spectrum and if we subtract those calculated with all the spectra, we can compare the residues for both designs. These residues for the apodized and the non-apodized designs are plotted in Figure 10. Between 5 and 95 mm, the differences for the apodized and the non-apodized designs were below 0.01%. The gap increased near the beginning and the end of the CFBG. This confirms that, theoretically, the continuous measurement of the shock or detonation wave-front was ideal over 90% of the CFBG length. Since the design without apodization was more sensitive (according to Figure 9), this design was selected for all experiments at CEA.

### 4.3. Experimental CFBG Response from the Initial Spectrum

**Figure 11:**
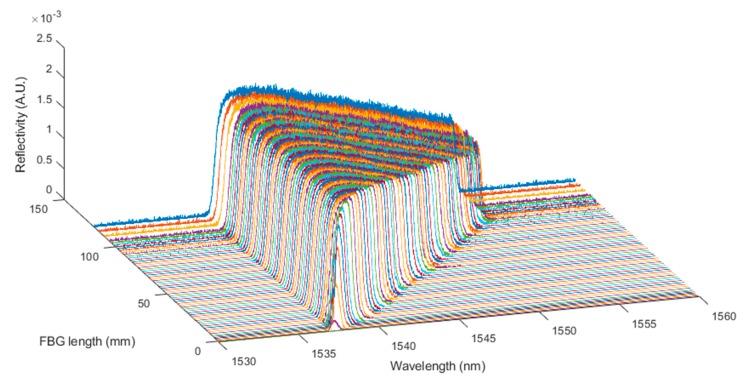
Normalized raw spectra measured during the shortening of a 100-mm CFBG.

The demonstration was done using a 100-mm CFBG centered at 1545 nm and with a 0.15-nm/mm CR. The design was with apodization, and the longer wavelengths were at the fiber’s end. This length is convenient to realize numerous optical characterizations at different dimensions. The CFBG was fixed on a motorized horizontal translation stage. A scalpel was placed on a manual vertical mount to cut the CFBG perpendicularly. During this experiment, the CFBG was connected to the BraggFast system and optical spectra were recorded, as a function of the horizontal translation stage position, using an OSA on the dedicated output (see Figure 3). In total, 64 spectrum measurements were recorded as shown in Figure 11. Firstly, 3-mm steps were taken to get closer to the CFBG edge; then, the steps were reduced to 2 mm until the end was almost reached. The last eight measurements were performed with a 1-mm step. Since the intensity of the ASE inside the BraggFast system fluctuated over time, we had to normalize all the curves between 1538.5 and 1541.5 nm.

**Figure 12:**
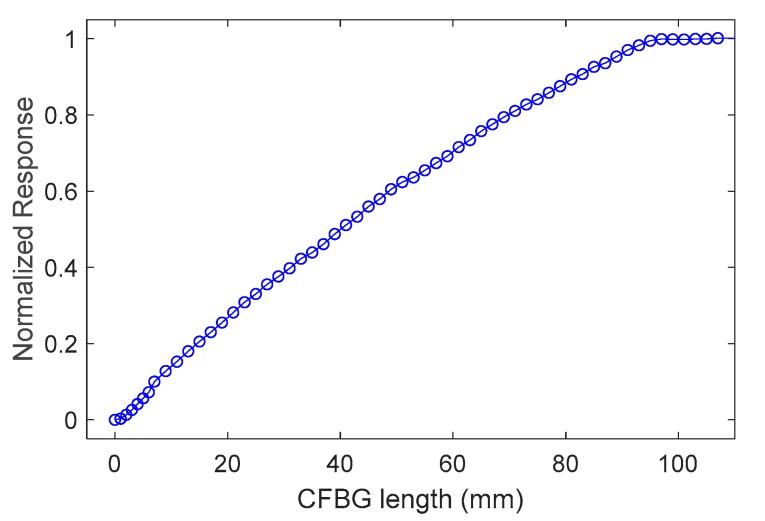
Experimental CFBG response as a function of the CFBG length calculated by integrating the spectra of Figure 11 one by one.

As for the simulations presented in the previous section, the experimental CFBG response was calculated by integrating the spectra of Figure 11 one by one. The initial spectrum amplitude was set at 1 for normalization purposes. The experimental CFBG response is plotted in Figure 12 as a function of the CFBG length. This CFBG was effectively shorter than the stated 100 mm (96 mm). The response was not as linear as in the simulations; this was due to the different spectral distortions from the source, the fiber component, and the CFBG itself. However, these spectral distortions were embedded in the initial CFBG optical spectrum.

**Figure 13:**
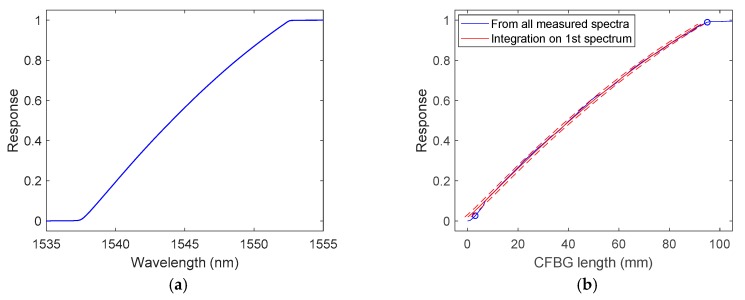
(**a**) Experimental CFBG response as a function of the wavelength by integrating the initial spectrum; (**b**) CFBG response as a function of the CFBG length calculated from the initial spectrum and fitted on the experimental CFBG response as a function of the CFBG length calculated by integrating the spectra one by one.

In order to verify this statement, the same demonstration as that in the simulation section is presented. To start, the initial spectrum was integrated as a function of the wavelength (see Figure 13a). The response increased with the wavelength because, as shown in Section 3, the longer wavelengths faced the shock or detonation wave-front at the end of the fiber. This curve was fitted between 2% and 98% in order to obtain the same CFBG experimental response as that obtained in Figure 12 as a function of the CFBG length. The fitted result is plotted in Figure 13b with 95% confidence bounds. The correlation coefficient was 0.9995, and the fitted CR obtained was (0.15602 ± 0.00020) nm/mm.

**Figure 14:**
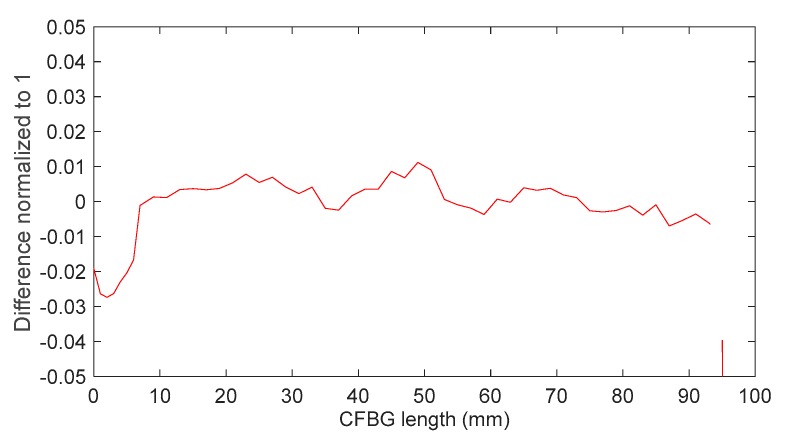
Difference between the CFBG response calculated from the initial optical spectrum and that calculated with spectra as a function of the CFBG length.

Differences between the CFBG response as a function of the CFBG length calculated from the initial spectrum and the CFBG response calculated by integrating spectra one by one are plotted in Figure 14. The levels were one order of magnitude higher than those obtained by simulations (see Figure 10) but the same conclusions can be made. The differences when the CFBG was much shorter certainly increased because the spectrum was too narrow to perform a proper normalization to compensate for the ASE power fluctuations over time.

The main conclusion is that we could obtain the CFBG response knowing its CR and the reflected spectrum before the experiment. The CR needs to be measured on each CFBG used in an experiment. The method is explained in Section 6.1 with experimental data for three types of CFBGs. This experimental demonstration to obtain the CFBG response from the initial spectrum and to measure the CR was also performed on CFBGs from another provider and for various lengths (18, 20, and 40 mm). The same conclusion was drawn.

**Figure 15:**
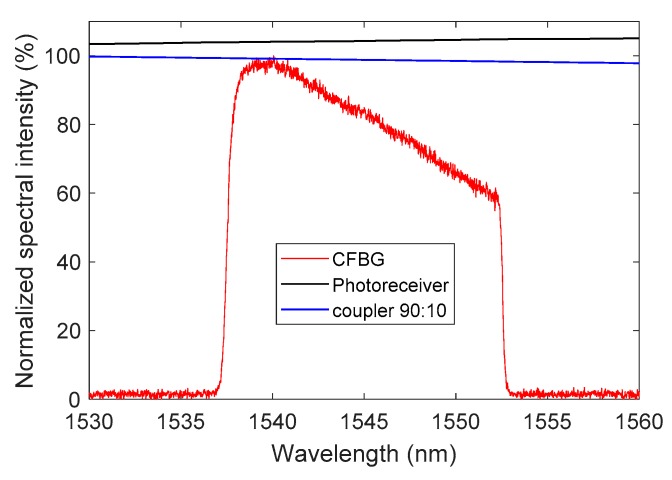
Normalized spectral response of the 90:10 coupler, the photoreceiver, and a 100-mm CFBG.

Smaller additional spectral distortions were taken into account in our BraggFast signal processing tool. The reflected optical spectrum was measured on branch other than the photoreceiver branch. The last 90:10 coupler did not have a constant ratio over the considered wavelength range, and the InGaAs photoreceiver itself is a well-known spectral response as a function of the wavelength. These parameters were almost negligible, but they were integrated in the calculation. Normalized spectral responses of the 90:10 coupler, the photoreceiver, and a 100-mm CFBG are plotted in Figure 15 for illustration.

## 5. Estimation of Experimental Uncertainties by Simulations

### 5.1. Definition of the Main Parameters

The method employed to obtain the X–T diagram from a BraggFast signal and a reference CFBG optical spectrum is described. In this section, series of signals are numerically generated with different noise levels and their impacts on the steady detonation velocity are evaluated. Listed below are the initial parameters for the simulations. SNR is the signal-to-noise ratio and FWHM is the full width half maximum.

- Steady detonation velocity: 7500 m/s;
- CFBG length: 50 mm;
- Oscilloscope sampling frequency: 1 Gs/s;
- OSA resolution: 0.05 nm;
- Standard deviation on the BraggFast signal SNR: 2%;
- Standard deviation on the optical spectrum SNR: 2%;
- CFBG FWHM: 20 nm;
- Upper CFBG response bound: 0.95;
- Lower CFBG response bound: 0.05;
- Number of points used to smooth the BraggFast signal: 7.

The CR can be calculated from the CFBG length and the FWHM (20 nm/50 mm = 0.40 nm/mm). The CR is very important as it directly links the detonation velocity to the change in wavelength in the CFBG, as shown in Equation (2). For convenience, we define the wavelength position factor (WPF) as the inverse of the CR. Then, WPF = 2.5 mm/nm in this numerical study.

(2) $$v\left(t\right)=\frac{\partial x\left(t\right)}{\partial t}=\frac{1}{\mathrm{CR}}\cdot \frac{\partial \left(\lambda \left(t\right)-\lambda_{0}\right)}{\partial t}=WPF\cdot \frac{\partial \left(\lambda \left(t\right)-\lambda_{0}\right)}{\partial t}.$$

**Figure 16:**
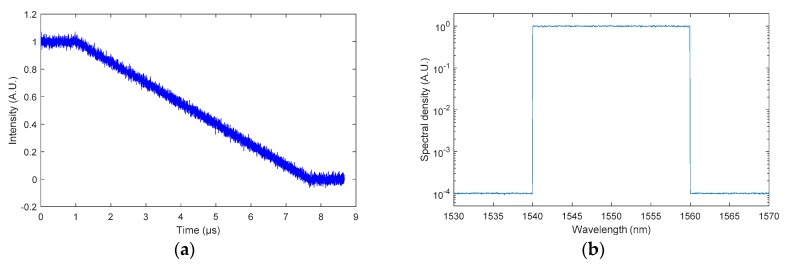
(**a**) BraggFast signal generated numerically with additional white noise in the case of a 50-mm-long CFBG and a detonation velocity of 7500 m/s. (**b**) Reflected optical spectrum generated numerically with noise of a 50-mm-long CFBG with 20-nm bandwidth (log scale).

Examples of the numerical generation of a BraggFast signal and a reflected optical spectrum with the parameters listed above are plotted in Figure 16a,b. The noise level of the BraggFast signal was constant from level “0” to level “1”. This white noise function simulated the noises of the ASE source, the photoreceiver, and the digital oscilloscope. The noise level for the optical spectrum was linear with the amplitude. This noise was only to simulate the ASE source noise since the OSA noise could be neglected. For this simulation, the background level of the optical spectrum was −40 dB and the maximum level was set at 0 dB.

### 5.2. BraggFast Signal Processing

**Figure 17:**
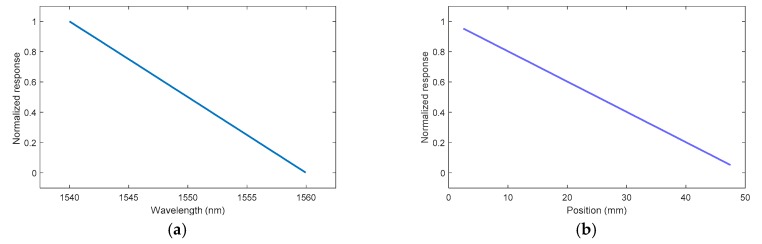
(**a**) CFBG response by integrating the optical spectrum as a function of the wavelength; (**b**) the same CFBG response but transposed to position using the wavelength position factor (WPF).

The first step was to calculate the CFBG response by integrating and normalizing the reference reflected optical spectrum (1). In practice, the minimum and maximum wavelength bounds are not obvious. Since the optical spectrum was measured using an OSA within a very high dynamic range (>50 dB), we set a threshold at −20 dB from the maximum reflectivity to determine the minimum and maximum wavelength bounds. The CFBG response as a function of wavelength is plotted in Figure 17a. The CFBG response as function of the position was obtained using the WPF. This curve was truncated at the upper and lower CFBG response limits (0.05–0.95) and then plotted, as shown in Figure 17b.

**Figure 18:**
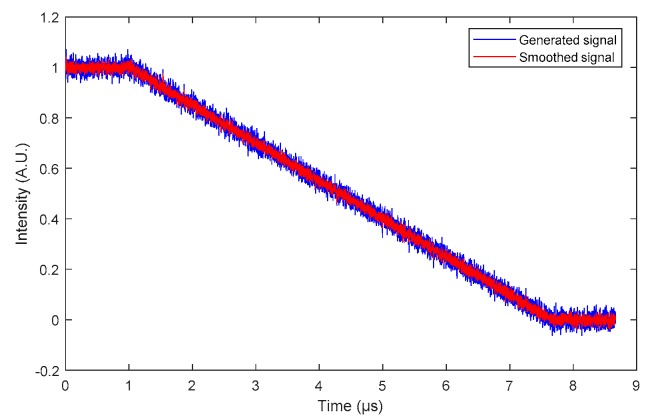
BraggFast signal smoothed with a Savitzky–Golay filter using seven sliding points.

The second step consisted of smoothing the BraggFast signal from the oscilloscope to slightly reduce the impact of the noise on the resolution of the steady detonation velocity value. In addition, in the case of an experimental signal, the upper and lower trace levels were normalized to “1” and “0”, respectively. The smoothing filter chosen used the Savitzky–Golay algorithm to more efficiently remove the points that were far away from the mean signal trace. An example of the filtering result is plotted in Figure 18 with seven sliding points.

### 5.3. Detonation Velocity Estimation

**Figure 19:**
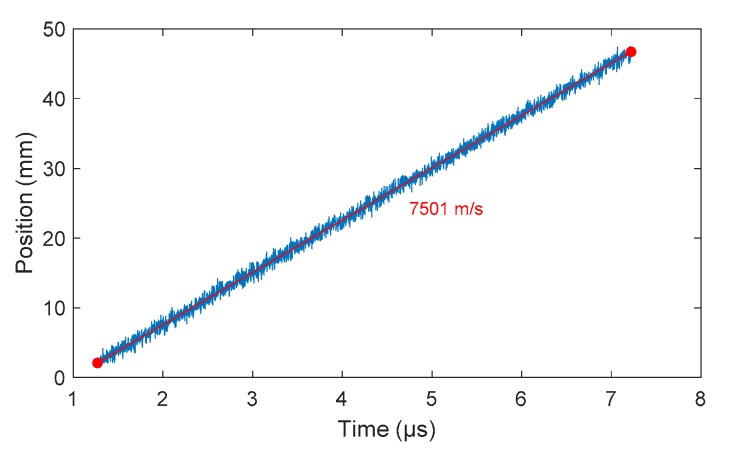
X–T diagram obtained from the smoothed BraggFast signal of Figure 18 and the CFBG response of Figure 17b. The detonation velocity value was obtained by fitting the data slope.

Once the CFBG response was calculated and the BraggFast signal was smoothed and normalized, the two curves could be compared to obtain the X–T diagram. For each point of the BraggFast signal between the upper and lower bounds (0.05–0.95), the time value was kept and the ordinate was interpolated on the CFBG response to obtain the corresponding position. To perform/carry out this interpolation, the abscissa and the ordinate axes were switched in the numerical function. The results of all these interpolations are plotted in Figure 19 as a function of the BraggFast time signal. In order to obtain the detonation velocity, a fit of the slope was performed using a standard least mean square method. The fitting slope is plotted in red in the same figure. Despite the high level of noise in the BraggFast signal and the optical spectrum, the detonation velocity value obtained, 7501 m/s, was very close to the input parameter given. The standard deviations are discussed below.

### 5.4. Influence of the Parameters on the BraggFast Signal Processing

**Figure 20:**
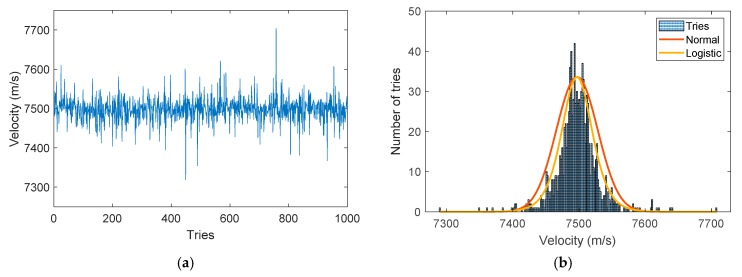
(**a**) Detonation velocity series with 1000 tries; (**b**) histogram of the series with fitted normal and logistic distributions.

The previous numerical steps could be repeated to obtain statistical information. Four series of simulations were carried out and analyzed to determine which parameter was the most relevant in the determination of the steady detonation velocity after signal processing. A study showed that, after 1000 simulations, the standard deviations of the tested distributions did not vary anymore. The four series of simulations were subsequently performed 1000 times. Using the parameters listed in Section 5.1, we obtained, from 1000 simulations, the steady detonation velocity series plotted in Figure 20a and the corresponding histogram plotted in Figure 20b with, on top, a normal and a logistic distribution.

For the normal distribution (Equation (3)), we obtained µ = 7497.16 and σ = 31.29.

(3) $$f\left(x|µ,\sigma \right)=\frac{1}{\sigma \cdot \sqrt{2\pi }}e^{\frac{-\left(x-µ\right)²}{2\cdot \sigma ²}}$$

For the less classical logistic distribution (Equation (4)), we obtained µ = 7497.06 and σ = 15.40.

(4) $$f\left(x|µ,\sigma \right)=\frac{e^{\frac{x-µ}{\sigma }}}{\sigma \cdot {\left(1+e^{\frac{x-µ}{\sigma }}\right)}^{2}}.$$

The logistic distribution is less common but it seemed to fit the shape of the histogram better. For both fits, the difference between the mean value and the theoretical value (|µ − V_th_|/V_th_) was less than 0.02%, which resulted in ± 1.5 m/s for a detonation velocity of 7500 m/s. Below this value in percentage, we would neglect the effect of the parameter tested compared to the impact of WPF. In the list of parameters, few had the same effect as the steady detonation velocity, the CFBG length, and the oscilloscope sampling frequency. In the end, they all changed the total number of points in the signal. Consequently, they did not need to be all run and the following series was tested:

- Series 1: CFBG length variation (10–100 mm);
- Series 2: number of smoothing points (3–20 points);
- Series 3: standard deviation in the BraggFast signal (0.01–3%);
- Series 4: standard deviation in the optical spectrum (0.01–5%).

The following conclusions could be made for each series:

In series 1, the difference between the averaged velocity value and the theoretical value decreased with the CFBG length from 0.015% at 10 mm to 0.005% at 100 mm, and the logistic standard deviation decreased from ~5 m/s to ~2 m/s. The conclusion is that the three parameters, namely, CFBG length, the steady detonation velocity, and the oscilloscope sampling frequency, had a statistically minor influence on the determination of the detonation velocity.

In series 2, more than seven points (at 1 Gs/s sampling rate) for smoothing the input BraggFast signal did not bring significant improvement in the determination of the detonation velocity.

In series 3, above 1% SNR, the signal processing underestimated the detonation velocity, and the standard deviation increased exponentially. This was due to the fact that, during the process of wavelength to position matching (described in Section 5.3), several points in the “1” or “0” level were wrongly taken into account. This can be resolved in several ways, e.g., by limiting the BraggFast signal to only the slope part, by increasing the number of points in the smoothing step, or by reducing the upper and lower bounds. Experimentally, the SNR was about 1% in the BraggFast signal. At this level, the logistic standard deviation was well below 10 m/s. Therefore, the influence of the SNR on the BraggFast signal could be neglected in the determination of the detonation velocity.

In series 4, we observed that the SNR in the CFBG optical spectrum had very little influence. This can be explained by the integration step used to normalize the CFBG response. This step leveled down the SNR in the CFBG optical spectrum. The influence of this SNR could be neglected in the determination of the detonation velocity.

This study showed that the uncertainty on the detonation velocity was mostly linked to the WPF. The other parameters were not so relevant through the signal processing in place. The main factors are then the standard deviation *σ_WPF_* (k = 1, normal law) on the WPF and the standard deviation during the slope fit of the X–T diagram *σ_Fit_* (k = 1, normal law). To conclude, the detonation velocity is given by Equation (5).

(5) $$V_{detonation}=V_{median}\pm 2\sqrt{\sigma_{WPF}^{2}+\sigma_{Fit}^{2}}\cdot V_{median}\;\;m/s\;(\mathrm{with}\;k=2,\;\mathrm{normal}\;\mathrm{law}).$$

In a more complex set-up like in SDT in a sphere, the X–T diagram does not necessarily provide straight slopes. In this event, three X–T diagrams are provided to take into account the standard deviation on the WPF (the most probable and the two extremes). Each of the three diagrams are smoothed, derived, and smoothed again to obtain shock/detonation velocity profiles as a function of time with boundaries.

## 6. Experimental Results

### 6.1. CFBGs Characterizations

**Figure 21:**
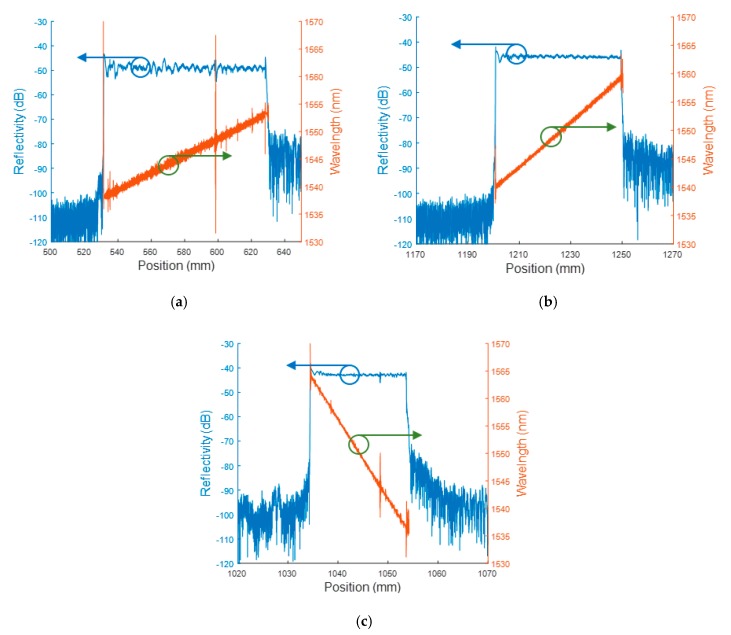
Optical frequency-domain reflectometry (OFDR) measurements of chirp rate (CR) for three CFBGs: (**a**) 100 mm long, CR = 0.1556 nm/mm; (**b**) 50 mm long, CR = 0.3981 nm/mm; (**c**) 20 mm long, CR = 1.4496 nm/mm with an inverted orientation.

As seen previously, the calculation of the detonation velocity is strongly dependent on the CFBG’s CR. Therefore, just prior to any experiment, the CRs of the CFBGs mounted on the set-up are measured using a commercial OFDR from Luna Technologies (OBR 4600). Several measurements are performed to decrease the measurement uncertainty on the CR. The results from a single CR measurement are plotted in Figure 21 for the three CFBG lengths typically used in our experiments (20, 50, and 100 mm). Few spikes were visible; these were minor local defects, which have no impact on the BraggFast measurements since the reflectivity does not drop with the position. The 100-mm- and 50-mm-long CFBG showed an increase in the wavelength with the position. We inverted this orientation for the shorter CFBG (20 mm long) so that it became more sensitive at lower shock pressure levels. Indeed, if the CFBG experiences a shock pressure which is lower than a detonation pressure, part of its spectrum is shifted toward the lower wavelengths [29]. Then, to avoid any overlap of wavelengths between the CFBG’s pristine section and the shocked section, the shorter wavelength side should be exposed to the upcoming shock front. In this configuration, the CFBG shocked section spectrum was most likely shifted outside the ASE optical spectrum; subsequently, the BraggFast signal recorded on the fast photoreceiver was the same as if the CFBG were destroyed. This approach was not applied to longer CFBGs as cladding-mode coupling losses start to play a significant role [30] and the CFBG’s spectrum would be too asymmetric.

OFDR measurements are typically repeated five times in the laboratory and 10 times in the field with a longer fiber (>20 m) between OFDR and the CFBG, to minimize the measurement uncertainties of the CR value. Standard deviations of about 0.15% were measured for the three types of CFBGs with at least 10 samples.

### 6.2. Steady Detonation Velocity on a Surface Line of a Bare Cylindrical Stick

#### 6.2.1. Set-Up

**Figure 22:**
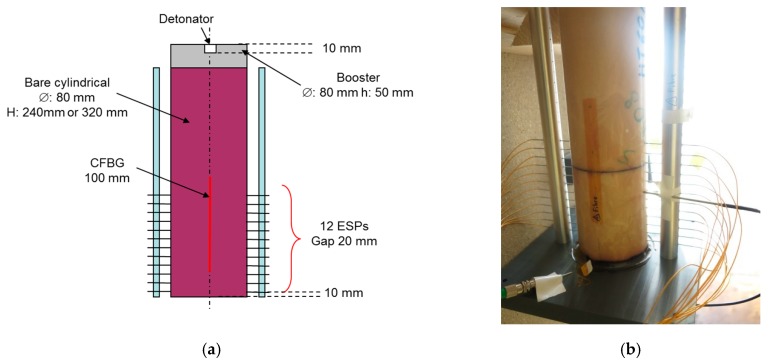
Bare cylindrical stick configuration: (**a**) set-up diagram; (**b**) image of an actual set-up.

In the following two experimental results, the explosive charges were bare cylindrical sticks, 80 mm in diameter and 240 or 320 mm long (see Figure 22). The explosive charge was initiated via a 50-mm-long HE booster with the same diameter. The detonator was inserted 10 mm deep inside the booster and centered along the symmetry axis. The steady detonation velocity was measured on the side of the explosive charge, the furthest away from the booster charge. The 100-mm-long CFBG (see Figure 21a) was pasted with a transparent tape on the cylinder surface parallel to the symmetry axis; its position was checked with a CMM (coordinate measuring machine) coupled to a camera. Currently, a V-groove is used to better position the CFBG and it is fixed with compatible glue. The CFBG angle position is not very sensitive; for instance, an error of 2° would lead to an error of less than 0.02% on the steady detonation velocity. In addition, 2 × 12 electrical short pin (ESP) sensors were placed on two surface lines of the explosive charge, as a reference measurement. In the following experiments, two different melt-cast HEs were tested: TNTO and Comp B. The TNTO composition was 59.5% NTO, 39.5% TNT, and 1% wax by weight, and the Comp B was 59.5% RDX, 39.5% TNT, and 1% wax by weight [25].

#### 6.2.2. Bare Cylindrical Stick Results with a CFBG on a Surface Line

##### TNTO Steady Detonation Velocity

**Figure 23:**
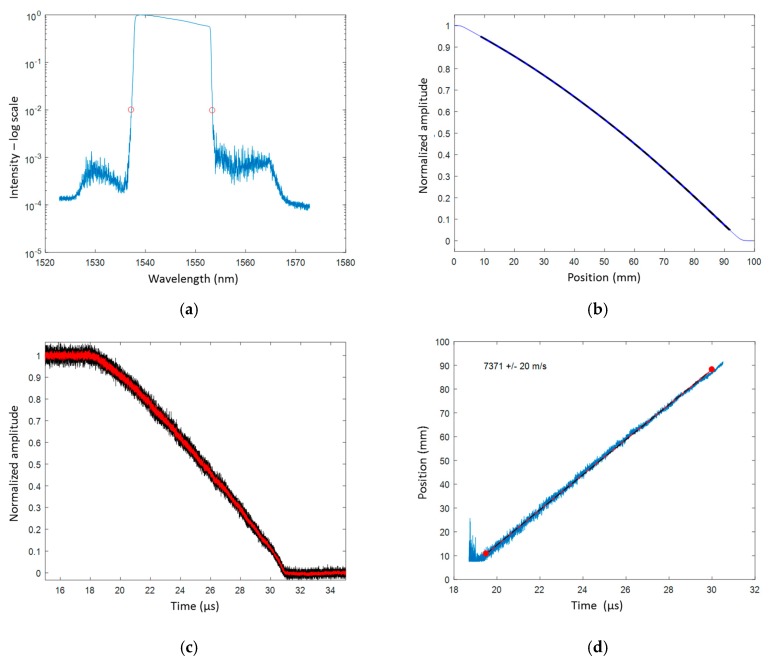
BraggFast signal: (**a**) CFBG reflected optical spectrum through the BraggFast system; (**b**) normalized CFBG response as a function of the position; (**c**) normalized and smoothed oscilloscope signals; (**d**) X–T diagram of the detonation wave-front with the fitted detonation velocity value.

**Figure 24:**
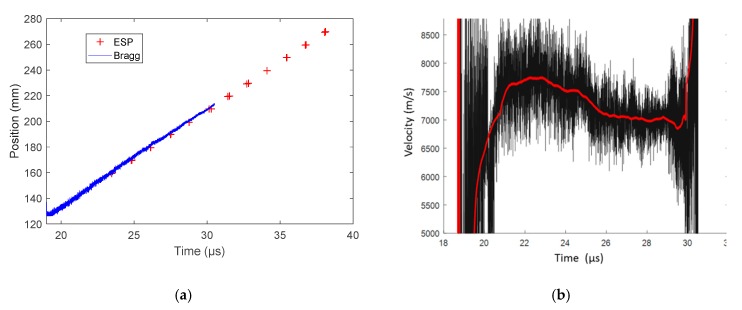
(**a**) X–T diagrams of the detonation wave-front measured by CFBG and electrical short pin sensors (ESPs): (**b**) smoothed detonation velocity derived from the CFBG X–T diagram.

Prior to the detonation test, the CR of the CFBG was measured, as well as the reflected spectra through the BraggFast system. The CR was measured to be 0.1631 nm/mm (±0.27%). The reference reflected optical spectrum of Figure 23a was integrated as a function of the wavelength and then transposed into position using the previously measured CR to obtain a normalized CFBG response, plotted in Figure 23b as a function of the position. In fact, three responses were obtained as we took into account the CR (the WPF) uncertainties (mean value, upper and lower bounds). After the experiment, the BraggFast signal processing could be carried out. The oscilloscope signal was normalized to “1” and “0” levels and then smoothed (Savitsky–Golay algorithm) over 20 sliding points, as shown in Figure 23c. Finally, this smoothed oscilloscope signal was compared with the CFBG responses to create the X–T diagram of the detonation wave-front, as shown in Figure 23d. The slope can be fitted to obtain the average steady detonation velocity. We obtained a velocity of (7371 ± 20) m/s between 19.5 and 30 µs. The ESP data provided an average velocity of (7536 ± 25) m/s between 23 and 39 µs, which was 2.2% higher than the velocity determined by the CFBG. Both datasets are shown in Figure 24a. In the CFBG signal, two slopes could hardly be seen; they were more obvious once the CFBG X–T diagram was derived to obtain the continuous velocity profile as a function of time (Figure 24b). Such a figure cannot be obtained with ESPs due to their limited number. The CFBG velocity profile data needed to be smoothed as the sampling rate was over 1 Gs/s. In the first area, between 21 and 25 µs, the average velocity was (7679 ± 21) m/s. This value was much closer to the ESP measurement result. However, in the second area between 25 and 30 µs, the detonation velocity was slightly lower (~7000 m/s). This could be due to the fact that the CFBG was not properly destroyed in the second zone, because the CFBG was not totally confined. This could be improved by placing the CFBG in a V-groove.

##### Comp B Steady Detonation Velocity

**Figure 25:**
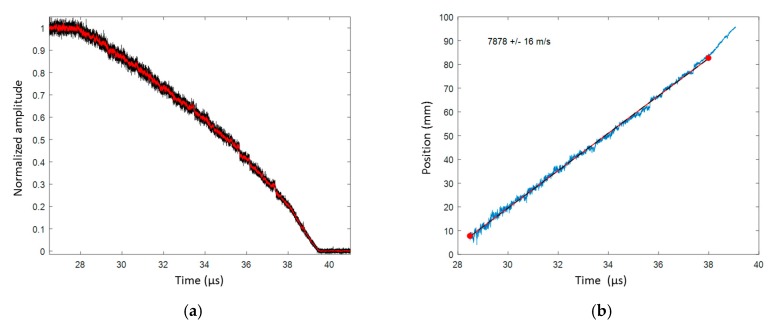
BraggFast signal: (**a**) normalized and smoothed oscilloscope signals; (**b**) X–T diagram of the detonation wave-front after signal processing with the fitted detonation velocity value.

**Figure 26:**
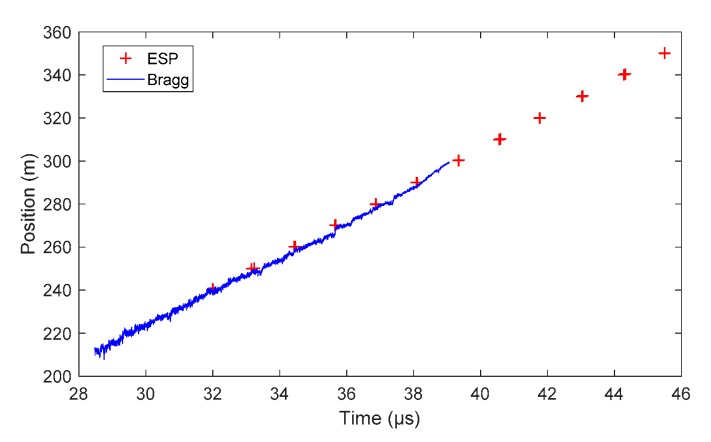
X–T diagrams of the detonation wave-front measured by CFBG and ESPs.

The same processing method as that described in the TNTO experiment was applied for the Comp B signal. The CR was measured to be 0.1544 nm/mm (±0.21%). The normalized and smoothed oscilloscope signals are plotted in Figure 25a, and the X–T diagram of the detonation wave-front after signal processing is plotted in Figure 25b. The comparison of the X–T diagram measured by the CFBG and ESPs is plotted in Figure 26. The average detonation velocity obtained by the CFBG data between 28.5 and 38 µs was (7878 ± 16) m/s, and the average detonation velocity obtained by the ESP data between 23 and 39 µs was (8133 ± 16) m/s, which was 3.1% higher. When looking more carefully at the oscilloscope data, one can notice numerous jumps, which indicates that the CFBG was not destroyed as linearly as it should have been. If we limited the calculation of the CFBG average detonation velocity between 30 and 32 µs, a value of (8162 ± 17) m/s was obtained. This value was much closer (−0.3%) to the ESP detonation velocity value.

### 6.3. SDT Characterization in a Wedge Configuration

#### 6.3.1. Set-Up

**Figure 27:**
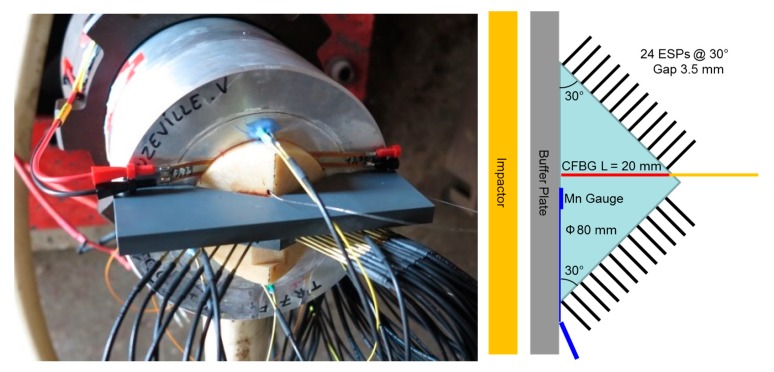
Picture of a typical wedge test set-up, with a sketch of a cross-section on the right.

The wedge set-up allowed the SDT to be studied. A double wedge-shaped HE charge was submitted to a planar sustained shock wave at different pressure levels using, in our case, a 98-mm-diameter powder gun. The experimental set-up is shown in Figure 27. The impactor and the buffer plate were chosen to ensure that the 30° base angle of the double wedge was sufficient to avoid any back release wave [25], which could adversely affect the SDT study. The input pressure was measured with a low-impedance (13 mΩ) Manganin gauge inserted at the interface between the buffer plate and the HE wedge base. The X–T diagram was measured using 12 ESPs placed on each wedge side. The position of each pin was precisely controlled with an uncertainty of about 30 µm using a CMM. In the first experiments, a 0.8-mm hole was drilled perpendicularly to position the 20-mm-long CFBG (see Figure 21c). Since the CFBG was only 125 µm in diameter, it was glued in a thin Teflon tube with an outside diameter of 0.7 mm. The assembly was also glued in the HE material wedge. The glue used was very fluid and was degassed properly. The curing time at room temperature took 24 h. In the most recent experimental set-ups, the hole was reduced to 0.5 mm and the CFBG was directly glued inside.

#### 6.3.2. SDT Results

##### TNTO SDT

**Figure 28:**
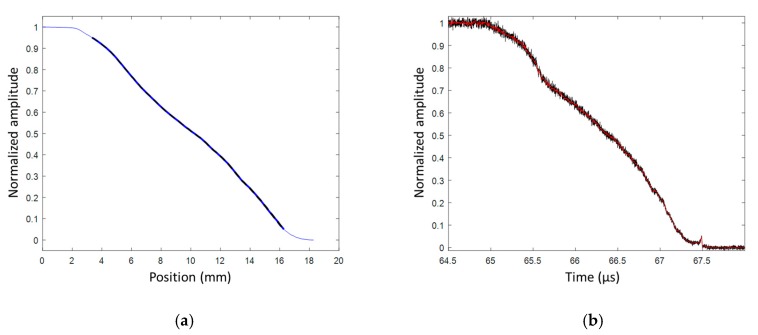
(**a**) Normalized CFBG response from the measured optical spectrum; (**b**) normalized and smoothed BraggFast signal.

**Figure 29:**
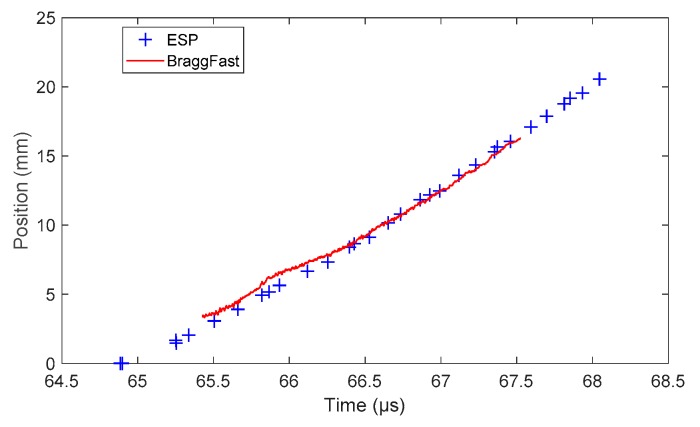
X–T diagrams of the detonation wave-front measured by CFBG and ESPs.

In this experiment on a TNTO wedge, the copper plate impactor was 96 mm in diameter and 10 mm thick. The aluminum buffer plate was 12 mm thick. The impact velocity was 1690 m/s. The CFBG was slightly shorter (16.5 mm) since the CFBG was cut to some extent to ensure that the grating started at the end of the fiber. In this experiment, the CFBG was glued in the wedge with a thin Teflon tube. The CR was measured to be 0.2190 nm/mm (±0.28%). The CFBG response calculated from the reference optical spectrum and the CR is plotted in Figure 28a. The shape was not a very straight line; this was mostly due to the ASE source spectrum. However, the measured BraggFast signal shown in Figure 28b had a similar shape. In the end, it did not affect the signal processing. The X–T diagram is shown in Figure 29 together with the ESP data. The steady detonation velocity determined with the ESPs between 66.25 and 68.05 µs gave a value of (7380 ± 10) m/s. The BraggFast signal seemed very close, between 66.5 and 67.6 µs; however, the fitted steady detonation velocity was only (6561 ± 20) m/s. A value of about 7400 m/s was only reached at the end of the CFBG. It is suspected that the CFBG was not sensitive enough with the Teflon tube. As a consequence, the CFBG response was delayed, and it slightly underestimated the wave-front detonation velocity.

##### HMX/PBHT (90%/10%) SDT

**Figure 30:**
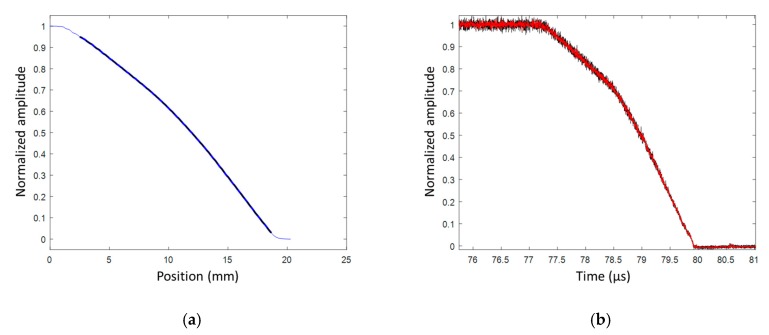
(**a**) Normalized CFBG response from the measured optical spectrum; (**b**) normalized and smoothed BraggFast signal.

**Figure 31:**
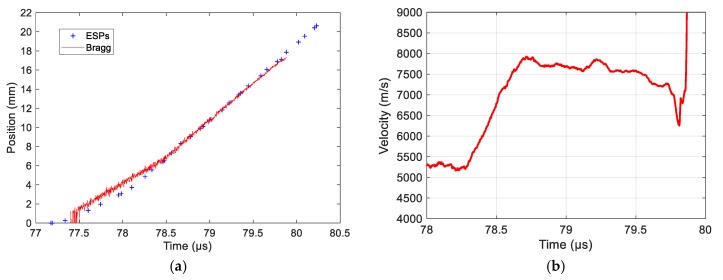
(**a**) X–T diagrams of the detonation wave-front measured by CFBG and ESPs; (**b**) wave-front velocity as a function of time measured by CFBG.

In this second experiment, the HE material studied was an HMX/PBHT composition with 90%/10% ratio by weight [25]. The aluminum plate impactor was 88 mm in diameter and 15 mm thick. The aluminum buffer plate was 5 mm thick. The impact velocity was 950 m/s. The CFBG was also slightly shorter (18.7 mm); thus, the grating was actually up the end of the fiber. In this experiment, the CFBG was glued in a 0.5-mm hole without any Teflon tube. The CR was measured to be 1.4349 nm/mm (±0.12%). The CFBG response calculated from the reference optical spectrum and the CR is plotted in Figure 30a. The measured BraggFast signal is shown in Figure 30b. The optical source of the BraggFast and the CFBG design were upgraded from the previous experiment; this is visible in the CFBG response, which had a smoother shape. The BraggFast signal showed two slopes as expected in an SDT experiment. This was confirmed in the X–T diagrams of the detonation wave-front measured by CFBG and ESPs, plotted in Figure 31a, as well as in the wave-front velocity profile as a function of time, plotted in Figure 31b.

The steady detonation velocity value calculated with ESPs between 78.5 and 80 µs was (7963 ± 73) m/s. Starting from the SDT transition point, the CFBG signal matched the ESPs data, and then a small delay was observed. As a result, the steady detonation velocity calculated from the BraggFast signal between 78.6 and 79.5 µs was (7726 ± 14) m/s, which was 3% lower than with the ESPs. Considering the wave-front velocity profile as a function of time plotted in Figure 31b, after 79.5 µs, a small decay in velocity was observed and led us to think that the CFBG was not properly broken by the wave-front. The glue layer (between 100 and 200 µm) around the CFBG might still have been too thick to obtain a reliable response. The velocity increased almost linearly from 5300 m/s at 78.3 µs up to 7700 m/s reached at 78.7 µs. During this acceleration phase, the HE material was more and more reactive. In this experiment, the run distance [25] was clearly visible, and a value of 8.0 mm was obtained.

### 6.4. Steady Detonation Velocity in a Sphere Configuration

#### 6.4.1. Set-Up

**Figure 32:**
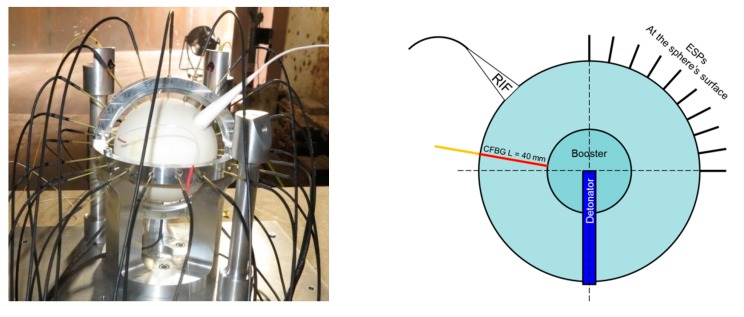
Picture of a typical sphere set-up, with a sketch of a cross-section on the right.

In this set-up, the HE charge had a spherical geometry in order to measure the sphericity of the detonation wave. In the center of this sphere, a smaller sphere was incorporated to act as a booster. This booster was initiated in its center by a cylindrical detonator, as illustrated in Figure 32. This detonator might create a small dissymmetry. ESPs were placed along two meridians to characterize the sphericity of the detonation wave. Nevertheless, the X–T diagram along a radius could not be measured with ESPs. However, this could be done with a CFBG if a small hole was drilled between the outer and the inner surfaces of the HE charge. In the following experiment, a 50-mm-long CFBG (see Figure 21b) was shortened to 40 mm to match the HE shell thickness. The hole diameter for the CFBG was 0.8 mm; therefore, the CFBG was glued with a thin Teflon tube following the same process as in the first wedge test (Section 6.3.1). A microwave interferometer (internally called RIF for radio interferometer) [31,32] with a 94-GHz frequency was also used. The microwave signal from the RIF was brought to the sphere through a dielectric waveguide (Teflon), and the output beam was expanded up to an 18-mm-diameter area through a cone directly glued on the outer sphere surface. The cone area on the sphere was intentionally flattened for this purpose. When the detonation wave-front propagates from the center of the HE material toward the surface, a material density gap associated with a permittivity gap at the wave-front interface reflected a fraction of the signal shifted in frequency by the Doppler effect. The reflected signal interfered with a reference signal at a fixed frequency, and the result was recorded in phase thanks to an in-phase and quadrature (I–Q) receiver. After processing the I–Q signals, the X–T diagram was obtained. The width of the microwave beam spatially averaged the position of the curved detonation wave-front. Even if the output from the RIF and the CFBG techniques was comparable, we expected the CFBG to be more sensitive to local effects than the RIF.

#### 6.4.2. RDX/PBHT (82%/18%) Sphere Detonation Results

**Figure 33:**
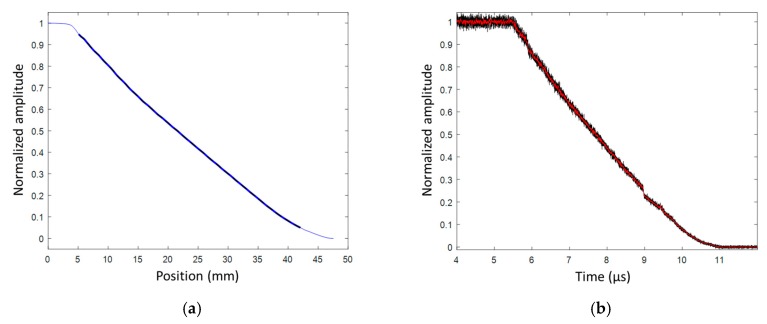
(**a**) Normalized CFBG response from the measured optical spectrum; (**b**) normalized and smoothed BraggFast signal.

**Figure 34:**
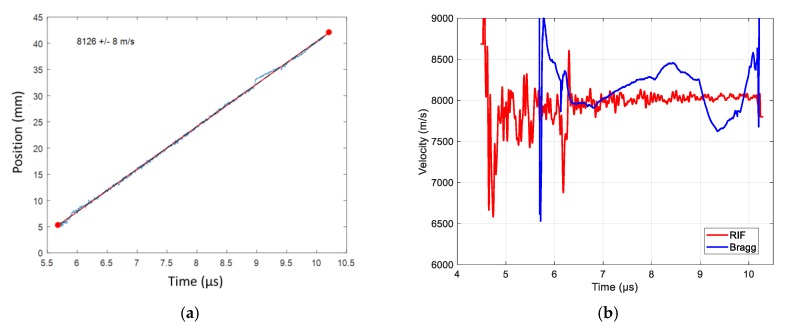
(**a**) X–T diagram of the detonation wave-front measured by CFBG with the fitted velocity value; (**b**) wave-front velocity as a function of time measured using the radio interferometer (RIF) system and the BraggFast system.

In the following experiment, the booster and the HE material of the study had the same RDX/PBHT composition with 82%/18% ratio by weight. The complete sphere weight (with the booster) was 1 kg, and the radius was determined accordingly. The CR of the CFBG was measured to be 0.4019 nm/mm (± 0.10%). The CFBG response calculated from the reference optical spectrum and the CR is plotted in Figure 33a. The measured BraggFast signal is shown in Figure 33b. The X–T diagram is shown in Figure 34a. The steady detonation velocity calculated between 5.6 and 10.2 µs was (8126 ± 8) m/s. The steady detonation velocity measured between 5.6 and 10.2 µs from the RIF data was 7990 m/s, which was 1.6% lower than with the BraggFast system. The velocity profiles could be calculated for the two systems with a smoothing step followed by a derivative step. The velocity profiles obtained are plotted in Figure 34b. According to the RIF data, the velocity increased from 7910 m/s at *t* = 6.5 µs to 8050 m/s at *t* = 9.9 µs. As a comparison, the BraggFast data showed a stronger acceleration between 6.8 and 8.4 µs. Later on, at *t* = 9 µs, the BraggFast signal showed a perturbation which depreciated the rest of the velocity profile. The CFBG did not average the wave-front position, as the RIF did, and it could have been influenced by local defects in the HE material.

### 6.5. Steady Detonation Velocity in Cast-Cured Bare Cylinders

#### 6.5.1. Set-Up

**Figure 35:**
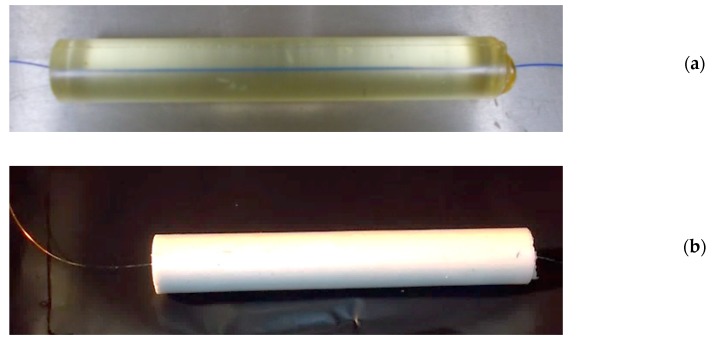
Pictures of (**a**) an inert and transparent cast-cured binder with a fiber inside, and (**b**) a cast-cured HE sample with a CFBG inside.

The objective of this set-up was, firstly, to remove the glue layer between the CFBG and the HE material and, secondly, to be able to insert inside the HE material a longer CFBG than feasible when a small hole is drilled. Cast-cured fabrication can allow a direct pouring of the uncured HE material around a CFBG kept straight in a cylinder mold on the revolution axis. It is not straightforward to keep the fiber straight during the curing phase; a dedicated process was created to ensure that the fiber was kept in position without being damaged by the holding system and by the stress undergone during the polymerization process. Specific containers were developed [15,16] at CRB. To observe the fiber position in the cast composition, the first experiments were performed with inert and transparent samples and fibers without any grating as shown in Figure 35a. Once the process was well established, real HE samples were produced, which incorporated a CBFG as shown in Figure 35b. In the next paragraph, detonation velocity results of an RDX/PBHT formulation with a ratio of 85%/15% by weight are presented. The sample had a diameter of 30 mm and a length of 150 mm. A 100-mm-long CFBG (see Figure 21a) was used, but it was unfortunately shortened during the handling or the processing steps.

#### 6.5.2. Detonation Velocity Results

**Figure 36:**
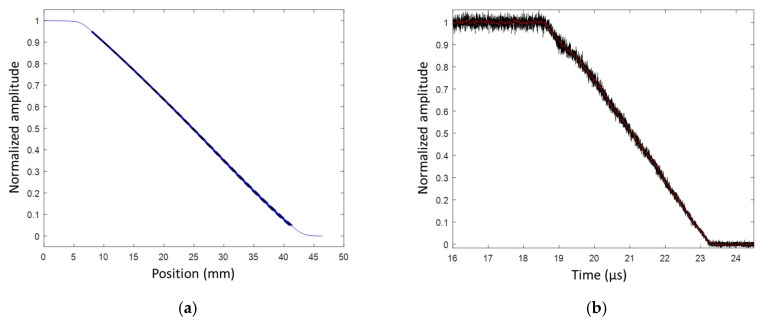
(**a**) Normalized CFBG response from the measured optical spectrum; (**b**) normalized and smoothed BraggFast signal.

**Figure 37:**
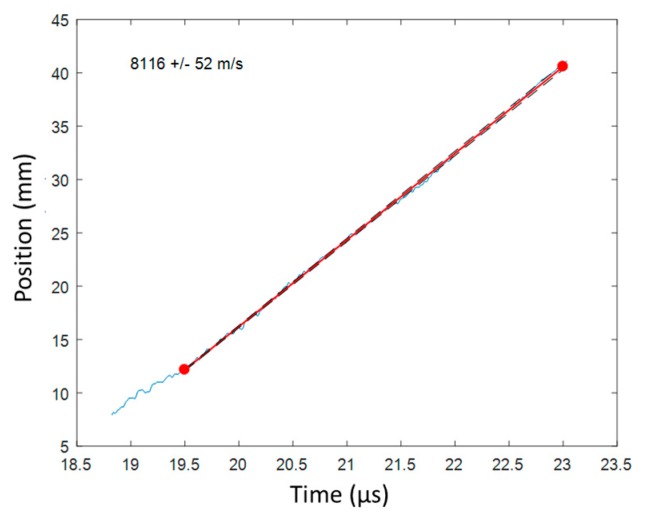
X–T diagram of the detonation wave-front measured by CFBG.

The CFBG CR value was measured by reflectometry to be 0.1854 nm/mm (± 0.64%) assuming that the optical effective index (*n_eff_)* did not change. The CFBG response calculated from the reference optical spectrum and the CR is plotted in Figure 36a. The measured BraggFast signal is shown in Figure 36b. The X–T diagram is shown in Figure 37. The steady detonation velocity calculated between 19.5 and 23 µs was (8116 ± 52) m/s. The steady detonation velocity measured on the HE surface by 10 ESPs was (8061 ± 121) m/s, which was 0.9% lower. The two values overlapped in their respective boundaries, which were, however, larger than in the bare cylinder stick experiments presented in Section 6.1. It would be interesting to repeat these cast-cured bare cylinder experiments with improved accuracy in the chirp measurement and with more ESPs. Furthermore, additional uniform FBGs could be added outside the BraggFast source spectrum to characterize the average strain [33] on all fibers and to possibly adjust the fiber optical effective index (*n_eff_)* used to measure the CR.

### 6.6. Detonation Curvature Profile in a Bare Cylinder Stick

#### 6.6.1. Set-Up

**Figure 38:**
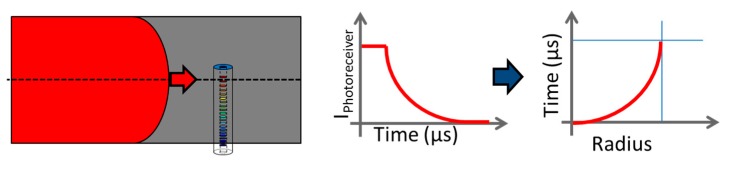
Detonation profile measurement in a cylinder using a CFBG. When the fiber is shortened by the detonation wave-front, the intensity of the reflected light decreases with time. This power variation is then converted into a change in position vs. time.

**Figure 39:**
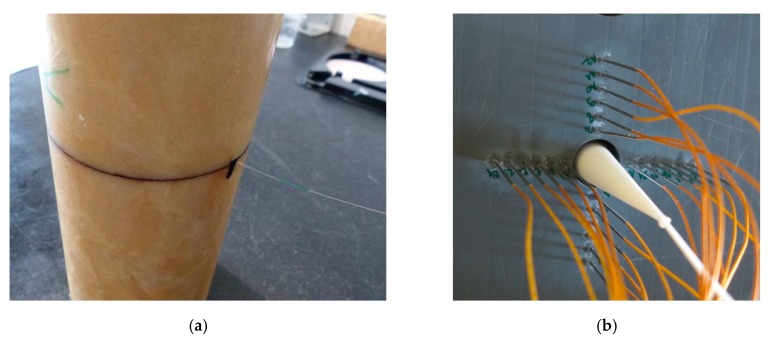
(**a**) Picture of an HE material cylinder with a CFBG placed perpendicularly to the cylinder axis to estimate the curvature of the detonation wave-front. (**b**) Picture of the cylinder end with 24 ESPs positioned in a cross shape. The white cone is connected to a microwave interferometer [31,32].

The BraggFast system could also be used to evaluate the curvature of the wave-front, as illustrated in Figure 38 and presented in Reference [17]. The CFBG is placed perpendicularly to the wave propagation and along a radius in the case of a cylinder. The CFBG can slightly cross the revolution axis to ensure a good sensitivity. Due to the detonation front curvature in such an unconfined cylindrical HE sample, the detonation wave-front first reaches the center of the cylinder and, later on, its edge. Knowing the steady detonation velocity with for instance a second CFBG placed on the side, the curvature of the detonation wave-front can be characterized. To easily incorporate the CFBG perpendicularly, the cylinder can be cut into two blocks and a small groove can be machined on one of the samples to align the CFBG before aligning and sticking together the two samples using the same fluid glue as in the wedge and sphere set-ups. A picture of such a set-up for Comp B (composition containing 59.5% RDX, 39.5% TNT, and 1% wax by weight) is shown in Figure 39a. The cylinder diameter was 80 mm, as in the bare cylindrical stick configuration described in Section 6.2. A 50-mm-long CFBG (see Figure 21b) was used to evaluate the curvature of the detonation wave-front. At the end of the cylinder, 24 ESPs were positioned in a cross shape, as shown in Figure 39b, while the white cone was connected to a microwave interferometer to measure the steady detonation velocity continuously (not presented here).

#### 6.6.2. Detonation Profile Results

**Figure 40:**
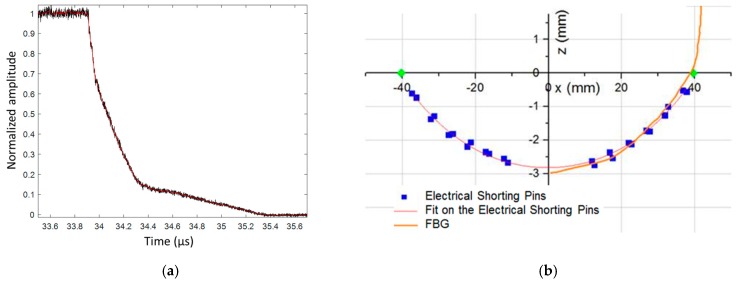
(**a**) Wave-front profile of normalized and smoothed signal; (**b**) experimental comparison of detonation profile measurements by ESPs and CFBG.

A 100-mm-long CFBG was used to measure the steady detonation velocity; a value of (7878 ± 16) m/s was obtained. The normalized and smoothed BraggFast signals, for the wave-front profile CFBG, are plotted in Figure 40a. The initial drop was very abrupt, as expected, from the top of the detonation wave-front. Then, the decay became slower and slower. At *t* = 34.3 µs, the wave-front reached the end of the CFBG at the side of the cylinder. Since the CFBG was longer than the cylinder radius, the end of the CFBG was destroyed in air at a much slower rate. After processing the signal, the fit, using a chain equation [34], over the ESP timing results was plotted and compared with the continuous measurement obtained with the CFBG, as shown in Figure 40b. The two datasets nicely overlapped with each other, validating our proof of concept.

## 7. Conclusions

In this paper, the use of CFBGs for shock and detonation velocity measurements was described in detail through the different sections. Firstly, a brief history of the development over the two last decades, together with the concept and a system design, was described. A wavelength on the CFBG corresponds to a position. The transposition from the wavelength domain to the distance domain is determined by the CFBG’s design, through the CFBG’s chirp rate (CR) value expressed in nm/mm. We also defined the wavelength position factor (WPF) (in mm/nm) as the inverse of this CR value because the uncertainties on this factor are directly proportional to the uncertainties on the steady detonation velocity. We showed that the CR value (and the resulting WPF value) can be measured by a commercial OFDR, in a nondestructive manner, for each CFBG mounted in a set-up. In order to better understand where the limits are in a CFBG response, a series of optical simulations were performed and designs with and without apodization were discussed. We concluded that a design without any apodization is preferred. The key step in such a measurement consists of the calibration of the CFBG response. A demonstration with optical simulations was carried out side by side with an experimental dataset to explain the calibration procedure using the initial reflected spectrum. The initial spectrum contained all the spectral distortion of the system which could, therefore, be compensated for. Thanks to the WPF, the CFBG response calculated from the initial spectrum could be expressed as a function of the position. Then, an estimation of experimental uncertainties on the steady detonation velocity was done by simulations with different parameters including several for the signal processing. It was demonstrated that the uncertainty on the detonation velocity was mainly linked to the WPF value. The other parameters tested appear to be insignificant through the signal processing in place. The main factors were then the standard deviation *σ_WPF_* on the WPF and the standard deviation of the slope fit of the X–T diagram *σ_Fit_*.

Once the method was clearly described, the results from seven experiments were presented for five different configurations and compared to other measurement systems. The bare stick cylinder configuration allowed steady detonation velocities to be characterized, and the wedge test was optimized to study SDT. A spherical configuration could also be used to study SDT but in a diverging spherical propagation mode. In order to have the closest possible contact between the CFBG and the HE material, cast-cured sticks were produced by directly pouring the HE material around a CFBG kept straight before and during the curing step. The last configuration evaluated the curvature of the detonation wave of a bare stick cylinder. In each case, the results were compared with ESPs except for the sphere experiment. In this set-up, a microwave interferometer with a 94-GHz frequency was used as a comparative measurement technique. To study SDT in a diverging spherical propagation using ESPs, a logosphere design [35] would be an ideal configuration.

One of the main conclusions of these detonation experiments is that CFBGs can efficiently be used to obtain a continuous X–T diagram of a shock/detonation wave-front. However, CFBGs are sometimes not sensitive over their entire length. A practical limitation is the ability to drill very narrow holes in the HE material over a long distance (>20 mm) and to efficiently fill them with a fluid glue to embed the CFBG. The compromise was found to be around 0.5 mm in diameter over a distance of about 30 mm. Any additional material to center the CFBG, such as a thin Teflon tube, should be avoided; otherwise, the CFBG response might be delayed or the CFBG may even be not destroyed efficiently by the detonation wave-front. An alternative method that was successfully tested was to directly cast the HE around the CFBG. This is effective but rather complex, and, in this configuration, we recommend placing additional monochromatic FBGs outside the BraggFast source spectrum to evaluate the average remaining strain in the CFBG after curing. The fiber effective group index value used during the characterization of the CFBG chirp may also need to be adjusted. CFBGs remain very flexible to obtain any X–T diagram in versatile set-ups. Their integration needs to be improved and better understood to increase the sensitivity. Therefore, new experiments were recently carried out using a dynamic dispersive spectrometer [36,37] in order to dynamically measure the entire reflected spectrum of the CFBG. Such a system was developed to measure pressure levels in inert and HE materials using monochromatic FBGs, but it can also detect any defect or delay in the shortening process of a CFBG during an SDT, for instance. Hydrodynamic simulations were also conducted to better understand the coupling of the shock between an HE material and the glass fiber [29].
